# Hair Cortisol, Perceived Stress, and the Effect of Group Dynamics: A Longitudinal Study of Young Men during Compulsory Military Training in Lithuania

**DOI:** 10.3390/ijerph19031663

**Published:** 2022-01-31

**Authors:** Rasa Smaliukienė, Svajone Bekesiene, Asta Mažeikienė, Gerry Larsson, Dovilė Karčiauskaitė, Eglė Mazgelytė, Ramutė Vaičaitienė

**Affiliations:** 1 General Jonas Zemaitis Military Academy of Lithuania, Silo 5a, LT-10322 Vilnius, Lithuania; svajone.bekesiene@lka.lt (S.B.); ramute.vaicaitiene@lka.lt (R.V.); 2 Department of Creative Communication, Vilnius Gediminas Technical University, Saulėtekio al. 11, LT-10223 Vilnius, Lithuania; 3 Microbiology and Laboratory Medicine, Department of Physiology, Biochemistry, Institute of Biomedical Sciences, Faculty of Medicine, Vilnius University, M. K. Čiurlionio st. 21, LT-03101 Vilnius, Lithuania; asta.mazeikiene@mf.vu.lt (A.M.); gerry.larsson@fhs.se (G.L.); dovile.karciauskaite@mf.vu.lt (D.K.); egle.mazgelyte@mf.vu.lt (E.M.); 4 Department of Security, Strategy and Leadership (ISSL), Swedish Defence University, Karlstad, Drottning Kristinas väg 37, 114 28 Stockholm, Sweden; 5 Department of Public Health, Inland University College of Applied Sciences, Hamarvegen 112, 2406 Elverum, Norway

**Keywords:** hair cortisol, perceived stress, accumulated stress, group dynamics, interpersonal cohesion, norm cohesion, task cohesion, military, decision tree, classification and regression tree (C&RT)

## Abstract

Previous research shows a nonlinear dependency between hair cortisol concentrations and perceived stress levels. This may be due to stress being targeted at the individual level despite it also being a social phenomenon which is often affected by group dynamics. Therefore, the objective of this study was to determine the influence of perceived stress on the hair cortisol level, considering the impact of the variables of group dynamics (interpersonal, task, and norm cohesion). Information was collected on 11 groups of, in total, 112 young men in three phases of time during their compulsory military training (covering nine months in total). The classification and regression tree (C&RT) method was used to predict hair cortisol concentrations in groups. The results show that the variability of the hair cortisol level in young men groups can be explained by perceived stress only when the groups were in formation process (47.7% normalised importance in Model 1) and when the groups were working on their final tasks (37.80% normalised importance in Model 3); meanwhile, the importance of perceived stress in explaining hair cortisol concentrations is low when the group is in a routine period of a group life-span (28.9% normalised importance in Model 2). Interpersonal cohesion (normalised importance 100% in Model 1 and 80.0% in Model 3) and task cohesion (normalised importance 78.6% in Model 2) were the most important predictors in the study area. These results point to the importance of the elements of group dynamics when it comes to explaining the nature of hair cortisol as accumulated stress biomarkers in young men.

## 1. Introduction

Compulsory military training provides a unique opportunity for research on young men’s resistance to psychological and physical stressors. Since a stressor is a situation which prompts an affective response [1]. Compulsory military training can be considered as being full of stressors: a tight military agenda, new peers, new tasks, and new activities. In these terms, the environment of compulsory military training involves not only the integration of an individual into the military environment. Conscripts live and perform in small groups with intense group dynamics: they cooperate, communicate, take leadership, and carry out other social interactions. All of these interactions ideally lead to high group cohesion, which is needed to carry out their military duties [2]. In principle, military activities are group-oriented [3,4]. Small-group dynamics strongly affect combat performance levels [5]. Consequently, group dynamics may explain the link between psychological and physical stress response, details which appear to be lacking in previous studies [6,7,8,9].

Using the theory of group dynamics [10], we aimed to extend the simple interrelationship model of cortisol and perceived stress which has been used in previous studies [6,7,8], and to include the variables of group dynamics which could better explain and predict cortisol levels. The research is important in terms of the fact that group dynamics is, to a high degree, a controllable process; the elements which are inherent in group dynamics can be influenced and changed, so by knowing the effect of group dynamics, we can develop military training programmes which train for stress resilience without significantly affecting the activation of cortisol secretion as a side effect of training.

### 1.1. The Effect of Group Dynamics on Stress

When considering stress as a ‘collective phenomenon which relies upon a team setting’ [11], group dynamics is an important variable that should to be taken into consideration when analysing stress in groups. Based on a group behaviour analysis [10,12], group dynamics is described as the development of a group which results from cohesion over the life-span of a group. According to McGrath et al. [13], group dynamics is an act which shows how a group emerges from the gathering together of several individuals. During military training, all inherent activities are based on small-group dynamics, the essence of which is to create a contextual environment which maximises the efficiency of such groups over a short period of time. With this in mind, a relatively short period of time—nine months in total—is designed into the Lithuanian armed forces in which young civilians should be transformed into a group of soldiers, i.e., small military units (squads), which are able to operate effectively in extreme conditions. This contextual environment is used to move a group through the various stages of group dynamics.

### 1.2. The Effect of Perceived Stress on Hair Steroid Hormone Cortisol Levels

An extensive body of literature documents the fact that stressors can affect the hypothalamic-pituitary-adrenocortical axis (HPA), the main physiological system which mediates the response to stress in the body. HPA regulates the synthesis and release of endocrine hormones, including its end product: cortisol, which is the primary hormone in the stress response system [14]. According to Balbo [15], cortisol causes a number of physiological changes during a stress response process. Although a momentary increase in cortisol secretion can play an adaptive role in a stressful situation [16], long-term oversecretion can have a negative impact on physical and psychological health [17,18]. Therefore, it is necessary to measure the cumulative levels of cortisol and to look for those stressors which have the greatest impact on its prolonged secretion.

Hair cortisol levels represent long-term (weeks to months) secretion [19,20] and the analysis of hair cortisol is increasingly being used in studies related to chronic stress [21]. However, the results of chronic stress studies are mixed, as the studies mainly show only the non-straightforward [22,23] relationship between hair cortisol variability and perceived stress levels.

### 1.3. Research Focus

In this study, we propose a novel approach when it comes to analysing the relationship between perceived stress and cortisol levels. Until now, research has sought a connection at the individual level, which contradicts the nature of stress if stress arises from interpersonal relationships. Therefore, our aim is to determine the influence of perceived stress on the hair cortisol level considering the impact of the variables of group dynamics (interpersonal, task, and norm cohesion). Taking into account the non-linear dependency between hair cortisol levels and perceived stress [6,7,24] and the social nature of stress [25], we hypothesise that both perceived stress and group dynamics influence the conscripts’ hair cortisol level and this impact varied throughout the entire period of compulsory military training. In this way, our research extends the current body of literature by using a longitudinal design to examine perceived stress and cortisol levels in individuals throughout the lifespan of a group.

## 2. Materials and Methods

### 2.1. Study Design

A longitudinal study was conducted on conscripts who had been formed into squads (involving groups of 9–11 people), covering the entire period of their compulsory military training (nine months). Hair samples and survey data were collected three times during this period: T1 took place after the first four weeks of training (the earliest possible time when the conscripts’ hair are long enough to collect); T2 took place in the middle of this training period (after 18 weeks); and, finally, T3 took place two weeks before the end of training (after 36 weeks), during which time the conscripts were highly involved in the final exercises and settlements.

Data were collected during the COVID-19 pandemic, when additional restrictions had been put in place in accordance with health and safety guidelines. Also, it should be mentioned that the second stage (T2) occurred during the lockdown period. Research was carried out using traditional paper-based questionnaires which were presented in the Lithuanian language.

The study was approved by the Vilnius Regional Biomedical Research Ethics Committee protocol, No 2020/10-1275-754. Each participant was provided with information about the study regarding their voluntary participation and the confidentiality of such participation. All participants provided informed written consent prior to any data collection taking place. Data which served to support the reported results has been archived in the ‘Lithuanian National Open Access Research Data Archive’ (MIDAS), at www.midas.lt. (accessed on 16 November 2021).

### 2.2. Biological Methods

The validity of measuring hair cortisol concentrations as a marker of chronic stress has been documented across studies [22,26,27,28] and has shown promising results compared to plasma or urinary cortisol levels [20]. Full details of the cortisol extraction procedure and analysis conditions using ultra-high-performance liquid chromatography-tandem mass spectrometry (UHPLC-MS/MS) system in this study have been reported elsewhere by Mazeikiene et al. [29]. For this study hair samples were taken from the posterior vertex region, as close to the scalp as possible. Each time, the samples were stored in foil at room temperature before being sent to the laboratory. Sunlight was not taken into account, as participants (soldiers) always wear headgear (berets or helmets) outdoors; hair samples were also not exposed to direct sunlight. Taking into account the fact that the conscript all had short hair, the hair cortisol level was determined from the first centimetre of scalp hair. This length represents approximately one month of hair growth and, accordingly, the amount of hormones which have been accumulated during the one month prior to analysis [30].

### 2.3. Psychological Measure

Perceived stress was measured using the 10-item perceived stress scale (PSS), which was developed by Cohen et al. [31] According to the meta-analysis by Cullen et al. [24], PSS is the most commonly used psychological stress scale in accumulated stress biomarker studies. To provide reliable and comparable results for this study, we also used the perceived stress scale as a psychological measure for the perceived stress assessment. The classic version of PSS measures involves stress levels over a period of one month, with this being fully consistent with our measurement of hair cortisol levels in one-month-long hair. The scale indicates low stress levels (with a score between 0–13), moderate stress levels (14–26), and high stress levels (27–40), as shown by Cohen et al.[31] An average Cronbach alpha for PSS is 0.865 (T1 = 0.853; T2 = 0.858; T3 = 0.885).

### 2.4. Group Dynamic Measure

Group dynamics were measured as group cohesion over the life-span of a group’s existence. In this study we measured group dynamics using the scale which had been developed by Ohlsson et al., [32] principally for research on multinational military staff exercises. The scale was modified and adopted to settings which were specific for the conscripts by adding items form Salo’s [33] research on Finnish conscripts, as well as items from the group cohesion scale which had been revised by Treadwell et al. [34] Three measures for group cohesion were composed:

- Interpersonal cohesion in team (CTE). This measure relates to interpersonal relations and an individual’s attraction to the group [35]. The measure was constructed using the 12-items inventory method which was measured by the Likert scale (with scores ranging from one, meaning ‘fully disagree’, to seven, meaning ‘fully agree’). The construct’s values vary in the interval (12–84), mean = 54. A sample item follows: ‘If a soldier fails during an exercise, the entire squad assists him’. The average construct’s Cronbach’s alpha for this study is 0.893 (T1 = 0.872; T2 = 0.880; T3= 0.928), which was similar to the team cohesion (bonding) inventory by Salo [33] which had a result of α = 0.86 [21], and which varied from 0.81 to 0.90 in the test by Treadwell et al. [34].
- Task cohesion (CTS). This measure shows how focused the group may be. It refers to an individual’s attraction to the group as a common interest towards the group task. The measure was constructed using task cohesion results following the aggregation of the eight items, as measured by the Likert scale (from one, meaning ‘fully disagree’, to seven, meaning ‘fully agree’). The construct’s values vary in the interval [8,9,10,11,12,13,14,15,16,17,18,19,20,21,22,23,24,25,26,27,28,29,30,31,32,33,34,35,36,37,38,39,40,41,42,43,44,45,46,47,48,49,50,51,52,53,54,55,56], mean = 36. A sample item follows: ‘Our squad finds non-traditional or innovative ways to achieve the set goal’. The average alpha value for CTS was 0.782 (T1 = 0.779; T2= 0.738; T3 = 0.830), which was very similar to the result gained by Ohlsson et al. [32] at α = 0.77.
- Norm cohesion (CIN). This measure mirrors the mainstream literature regarding small group research within the military in which ‘military cohesion’ is used as a general term to describe microlevel dynamics amongst soldiers, and which leads to combat efficiency [36]. The measure was constructed using the 6-items scale as measured by the Likert scale (from one, meaning ‘fully disagree’, to seven, meaning ‘fully agree’). Construct values vary in the interval [6,7,8,9,10,11,12,13,14,15,16,17,18,19,20,21,22,23,24,25,26,27,28,29,30,31,32,33,34,35,36,37,38,39,40,41,42], mean = 27. A sample item follows: ‘The squad seems to be aware of the group’s unspoken rules’. The average construct’s Cronbach’s alpha is 0.884 (T1 = 0.869; T2 = 0.878; T3 = 0.904).

### 2.5. Groups

Following the recommendations for group dynamics studies [37], individual data needs to be nested into groups within a higher-level unit (in our case this is squads). Following this recommendation, the group variable (G ID) is not a measure in our study but is an indicator for group segmentation. The nested data means that individual units are not independent [37], while G ID represents the group itself and helps to group the data for other measures.

### 2.6. Participants

A random sample of 11 groups (squads) from one military battalion from the Lithuanian Armed Forces were selected for this study. For military training, only mentally and physically healthy youth are recruited, therefore the research sample was comprised of young and healthy men. The following exclusion criteria were: usage of synthetic glucocorticoids, medications and chemically colouring hair in the last three months. Anyone who refused to participate was also withdrawn from the study. Female conscripts were excluded from the statistical analysis of this study due to their small number. After removal had taken place, the analysis was conducted in the eleven groups, *n* = 110 for the first stage, *n* = 106 for the second stage and *n* = 107 for the third stage (in total *n* = 112).

Participants were aged between 18–26 years (M = 20.32 years), and all were male. The majority of the sample had secondary (or unfinished secondary) education (89.5%), prior to the commencement of their conscription training, and the majority had a job (56.6%), and lived with their parents (67.3%). Detailed information on demographic and anthropometric data for each of the eleven groups is presented in Table 1.

### 2.7. Methods of Statistical Analysis

All of the analyses were carried out using the IBM SPSS Statistics 27v software. At the beginning we assessed data using descriptive analysis. Data for hair cortisol levels were normalised using logarithmic transformation to normally distribute steward data. Repeated-measures MANOVA was used to examine the differences for repeated measures of hair cortisol and the Bonferroni’s pairwise comparison test [38] was conducted to test significant differences between the mean of the study variables. The hypothesis of data sphericity was tested using the Mauchly and the Greenhouse-Geisser adjustment tests [38]. Mean differences were tested using Friedman’s tests for variables that violated the assumptions of normality [40].

Correlation analysis served to evaluate the relationship between hair cortisol levels and self-reported data. Following this, we applied the C&RT algorithm and created decision trees to identify the relationship between hair cortisol levels and other variables in the conscripts’ groups for each time point. Decision tree modelling includes data which were measured on different scales [41,42] based on its non-linear relationship [35,36] and produces subsets of the data which are as homogeneous as possible with respect to the target variable [44]. The score of the best predictors for the division of subsets was calculated using the least squared deviation value, which indicates the improvement measure attributable to each of the four exploratory variables used in this study. The score obtained is reported as the improvement in the decision tree modelling results [45]. Thus, the main purpose behind the use of the decision tree in our analysis is to achieve a more concise and perspicuous representation of the relationship between an observable variable (hair cortisol levels) and explanatory variables.

As for the technical conditions which have been specified in the execution of the designed models, the minimum *p*-value which was required for splitting and merging was set to 0.05, with a minimum number of records in the parent branch of fifteen, and a minimum in a child branch of five. Since the tree growth algorithm treats all variables either as categorical or ordinal, no standardisations were required for this step.

## 3. Results

### 3.1. Data Assessment

Table 2 presents the descriptive statistics of five variables which were repeatedly used in the analysis, to a total of three times (T1, T2, and T3).

**Figure 1:**
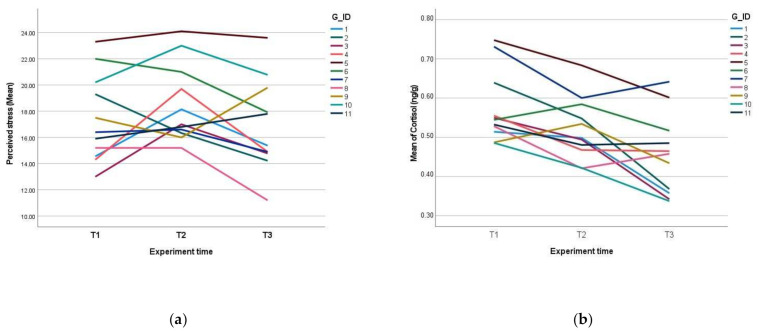
The degree of perceived stress and hair steroid hormone cortisol levels in groups during three time points (T1, T2, and T3): (**a**) perceived stress is measured using the perceived stress scale, in which a score of between 14–26 indicates moderate stress levels; (**b**) hair steroid hormone cortisol levels (ng/g).

During the second time point in the study (T2), changes in the hair hormone cortisol levels remained on a downward trend. The means for perceived stress and hair cortisol in groups are shown in Figure 1.

The Pearson correlation analysis indicates strong and statistically significant relations between variables in the self-reported data (involving perceived stress and group dynamics variables), and a weak relationship between hair cortisol levels and other variables (Table 3). At the beginning of the training period (T1) and at the end of it (T3) there were no significant correlations to be found between hair cortisol levels (COR) and perceived stress levels (PSS); and only in the middle of the training period (T2) were perceived stress and hair cortisol levels likely to be weakly correlated (r = 0.208, *p* = 0.005). The correlation between perceived stress and indicators of group cohesion was significant at all three time points, and this even increased towards the end of the training period, when the correlation coefficient became greater than 0.5 in all three cohesion cases: interpersonal (CTE), task (CTS), and norm (CIN).

To find out whether there are statistically significant differences between hair cortisol measurements, a repeated measures MANOVA was used to compare differences in hair cortisol levels (ng/g) in a repeated manner (T1, T2, and T3). The results showed statistically significant differences between hair cortisol measurement according to three time point measures (Greenhouse-Geisser F (1.904) = 13.826 *p* < 0.001). Pairwise comparisons by the Bonferroni test led us to realise that the cortisol level at the end of service (COR_3; M = 0.461) differ significantly from the cortisol level at the beginning of service (COR_1; M = 0.569; *p* < 0.0001) and from the cortisol level in the middle of service (COR_2; M = 0.528; *p* < 0.0001), but there were no statistically significant differences between the cortisol level at the beginning of service (COR_1; M = 0.569) and in the middle of service (COR_2; M = 0.528; *p* = 0.222). The detailed results of the pairwise comparisons are presented in Table A1 (Appendix).

The Friedman test was performed to examine statistically significant differences between three study points (T1, T2, and T3). The results of the tests showed differences for interpersonal cohesion (CTE1, CTE2 and CTE3; χ2(2) = 22.765 *p* < 0.0001), for task cohesion (CTS1, CTS2 and CTS3; χ2(2) = 13.286 *p* < 0.001) and for norm cohesion (CIN1, CIN2 and CIN3; χ2(2) = 15.960 *p* < 0.0001). However, no statistically significant differences were found between of perceived stress levels at the different time points (PSS1, PSS2, and PSS3; χ2(2) = 5.913 *p* = 0.052).

### 3.2. Model Specification

In total, three decision trees were computed, one for each time point. A variable group (G_ID) was used as the first (forced) variable to nest the individual data into groups (the squads) in each model. The constructed models used the same variables during different time points and the results on the constructed decision tree models are presented in Table A2 in the Appendix.

Model 1 represents the variation in hair cortisol levels at the beginning of training period (T1) (see Table A3 in the Appendix). The modelling revealed that at the beginning of the training groups were almost homogeneous according to the distribution of the cortisol level: 9 of 11 groups (in total 81.3 % of the conscripts) were nested in one node (Node 2) with an average cortisol level of 0.5292 ng/g. Following further results from the modelling, low task cohesion (CTS1, with an improvement value of 0.003) contributes to the prediction of high cortisol levels for six conscripts (at 0.734 ng/g), and to separate them from the groups in which they were located (see terminal Node 3). Furthermore, high hair cortisol levels (at 0.701 ng/g) were predicted for those conscripts who perceived average or high levels of stress (PSS1 >25.5, Node 6). On the contrary, the lowest hair cortisol level (on average 0.3616 ng/g) was predicted where task cohesion was higher than average (CTS1 > 29.0) following the moderate to low level of perceived stress and interpersonal cohesion (PSS1 ≤ 25.5; CTE1 ≤ 61.5)

Model 2 represents the middle of the training period (T2). It is somewhat different from that for the beginning of the training period (Table A4 in the Appendix). Model 2 revealed five groups (covering a total of 47.5% of conscripts) which could be nested together according to their homogeneity when it comes to predicting average hair cortisol levels (at 0.5860 ng/g). The second branch (with 52.5 % of conscripts) is divided by task cohesion (CTS2) and indicates that higher task cohesion leads to lower hair cortisol levels (at 0.4112 ng/g). The lowest average cortisol level (0.366 ng/g) was predicted where task cohesion (CTS2) was below average.

Model 3 represents the end of the training period and indicates two almost equal branches (Table A5 in the Appendix). The first branch represents the situation for slightly higher hair cortisol levels (at 0.588 ng/g for 28.2% of conscripts) associated with average to high levels of perceived stress (PSS3 > 17.5). The second branch represents lower hair cortisol levels (at 0.391 ng/g) associated with an average to low level of perceived stress (PSS3 ≤ 17.5) and interpersonal cohesion (CTE3 > 62). Furthermore, the lowest hair cortisol levels in our study (at 0.230 ng/g,) were associated with low to average norm cohesion (CIN3 ≤ 31.5), and average to high interpersonal cohesion (CTE3 > 41.0).

It is found that at the beginning of service (T1) the normalised importance value for perceived stress is at the highest level when compared with other time periods (Table 4, Model 1); that is, it has the biggest influence in predicting the hair cortisol level. Additionally, it is observed that at the beginning of service (T1; Model1) interpersonal cohesion (CTE1, NIMP = 100%) and task cohesion (CTS1, NIMP = 59.2%) are the strongest predictors of hair cortisol levels, more than perceived stress (PSS1, NIMP = 47.7%).Very similar results were obtained in Model 2, where task cohesion (CTS2, NIMP = 78.6%) and interpersonal cohesion (CTE2, NIMP = 32.4%) are stronger predictors than perceived stress (PSS2, NIMP = 28.9%). Two other indicators (CTE3 and CIN3) serve to better explain hair cortisol levels in Model 3 than perceived stress (PSS3, NIMP = 37.8%). According to the results, perceived stress and all three variables of group dynamics (interpersonal, task, and norm cohesion) are included in the static models. However, the perceived stress level is statistically important (NIMP is greater than 45%) only at the beginning (Model 1) and at the end (Model 3) of training. The importance of variables of group dynamics varies between the time points of compulsory military training.

## 4. Discussion

In line with previous studies on the interrelationship between perceived stress and hair cortisol levels in young, healthy male groups during military training [44], in our study we identified only a weak correlation between hair cortisol level variability and perceived stress, and only then at certain time points in terms of group dynamics. As suggested by Kozusznik and Euwema [45], the significant relationship between perceived stress and hair cortisol occurs only when stressors are perceived. In steady circumstances the concordance between psychological stressors and hair cortisol is weak in healthy individuals according to the meta-analysis which was handled across a total of 213 studies by Cullen et al. [24].

The decoupled nature of perceived stress and hair cortisol levels which has been found in previous studies [44], and also in our study, indicates that the change patterns in terms of hair cortisol are different than the changes in terms of perceived stress during military training. In a new environment, hair cortisol levels consistently decreased over time as the group members adapted. Being in and working in a group strengthens the resilience of group members to stress as group cohesion rises over time. For example, Williams et al. [48] found in their research on military training that group cohesion determines the psychological health of new soldiers, as a positive social climate in the group plays a protective role in the well-being of the group members. This is what our study shows when hair cortisol levels fall with some statistical significance in terms of comparing the first and last time points of military training (with the the Bonferroni’s pairwise comparison test result of *p* < 0.000) over the nine months of conscription training. This finding is consistent with previous studies which have found that hair cortisol levels decrease in a constant environment even where stressful interventions are involved [49]. To sum up, this study confirms the findings of previous studies in terms of the decoupled nature of perceived stress levels and of hair cortisol levels, despite the fact that hair cortisol levels are agreed to be a biomarker of accumulated stress.

This study examined the role of perceived stress and the perceived elements of group dynamics when it came to predicting hair cortisol variations. Our findings confirm that the chosen aspects of group dynamics contribute significantly to this prediction as stress is experienced in the group. Specifically, interpersonal cohesion in a team was the most important element of the group dynamic during the first and last stages of group development (100% and 80% of normalised importance for CTE1 and CTE3 respectively was presented by Model 1 and Model 3). According to the intensity of activity, the first and last stages (T1 and T3) in our research were probably the most stressful for conscripts because of task intensity and, as noted by West et al. [50]: ‘hormonal responses to exercise are significantly dependent upon the relative intensity of the activity in question’. In our study, interpersonal cohesion explained the hair cortisol levels. Very similar results were found by Field et al. [51], in terms of saliva cortisol levels, where peer support contributed a statistically significant decrease in cortisol levels in a non-military setting. In the military, interpersonal cohesion in a group makes an even bigger impact. As shown by Brailey et al. [52] in relation to military deployments, when group cohesion is growing, group dynamics have been found to produce a decreasing effect even for ‘past stressor exposures and post-traumatic stress disorder symptoms’ [52]. With our findings we can extend and specify these conclusions, by adding that interpersonal cohesion becomes the most important element in the hair cortisol prediction model when groups were under the process of formation and when groups were working on their final tasks.

In the middle of the compulsory military training period, when routine tasks and activities are being carried out and the level of newness is decreased, task cohesion (78.6% of normalised importance for CTS2 in Model 2) is the most important predictor in the study area. Task cohesion in a group encourages group members to work for the benefit of the group rather than for themselves. This is referred to as ‘social fitness’[53], encouraging collaboration and collectivism which increases group stress resilience. With our findings we can extend and specify these conclusions, by adding that task cohesion becomes the most important element in the hair cortisol prediction model in the middle of the compulsory military training period, when routine tasks and activities are being carried out.

The results of previous studies indicate the importance of multiple variables in predicting hair cortisol levels, or as it was concluded by O’Brein at al [22], the level of hair cortisol does not always have a direct relationship with ‘single stress indices. It was found that the hair cortisol level can be predicted by the perceived stress level in association with dispositional optimism [53], resilience [54], and high workload [26]. Our results add the variables of group dynamics to this list. Group dynamics variables (interpersonal, task, and norm cohesion) along with perceived stress can be used to predict hair cortisol levels. It is important to note that the effects of group dynamics variables change over the life-span of a group; therefore, the results of this study are in line with the theory of group dynamics [13] and studies that emphasise changes in group cohesion over time [55].

To our knowledge, this is the first study to investigate the interrelation between hair cortisol levels using nested data within servicemen’s groups. We observed a considerable effect evidenced by group dynamic variables (interpersonal cohesion, task cohesion, and norm cohesion) in predicting averages of hair cortisol levels in groups. Our study provides evidence that, when groups emerge and when groups are dealing with final challenging tasks, interpersonal cohesion can statistically increase the accuracy of the prediction while assessing the impact of perceived stress on the hair cortisol levels. This is a promising result, one which tends to indicate that, by controlling elements of group dynamics, we can develop military training programmes which train conscripts for stress resilience without significantly affecting the activation of cortisol secretion as a side effect of training.

Several limitations in this study should be addressed. Firstly, our research took place during the COVID-19 pandemic, with social distancing measures between and within military units being applied. This may have become an additional stressor which served to influence hair cortisol levels and perceived stress measurements, as has been shown in previous studies [56,57]. The results of the second phase (after four months of training) of our study and of Model 2 should be interpreted with caution, as the information contained there was collected during the national COVID-19 lockdown and it was not possible to have a control group to evaluate this effect. However, compared to a very similar pre-COVID-19 study composed of Swiss conscripts [46], the dynamics of stress level indicators are very similar during the first four months: perceived stress increased while no significant differences were found between the level of cortisol at the beginning of training and after four months. This implies that the dynamics of stress indicators among young men during basic military training are more dependent on the stressors of the military training itself that the additional restrictions applied during pandemic. However, an additional longitudinal study in the non-COVID-19 period is required to confirm this hypothesis. Therefore, we plan to continue this longitudinal research with a new group after the pandemic is over.

Secondly, the first hair sample collection took place after the first four weeks of service because the short hair of the conscripts had to grow to the length required for the examination. For this reason, the study does not show cortisol levels at the time point when the conscripts entered the new military environment, which limits the broader interpretation of the results of this study. Thirdly, our research sample was comprised only of men. Although we purposefully selected only men for our study to avoid the impact of gonadal hormone levels which can be caused by premenstrual changes in women [58], this choice served to restrict the interpretation of the results in the population of young men. Fourthly, we have to take into consideration the country factor as a research limitation, as the research was carried out only in Lithuania (in the Europe’s north-eastern corner). This could result in some variations in hair cortisol levels determined by geographic and cultural environments. In previous studies, various hair cortisol levels were identified when comparing young adults from different countries, and a higher level of hair cortisol was identified amongst Northern Europeans when compared to the results for Southern Europeans [59]. As noted by Wester et al. [60], these differences are determined by different levels of sun exposure on the human body in distinct geographical regions in general and on hair cortisol levels in particular. Finally, despite the fact that the mean level of hair cortisol is widely used as a chronic stress biomarker [22,27,47,61], it is essential to indicate that different modifications of liquid chromatography analysis are used to detect hair cortisol concentrations. Optimisation and standardisation of the extraction and quantification of cortisol content is still required [20]. Due to existing methodological modifications and protocol variations, a unified range of reference values has not yet been established and can be indicated as a limitation of hair cortisol concentration measurement in chronic stress research.

## 5. Conclusions

The results show that hair cortisol level variability in young men groups can be explained by perceived stress level only when groups were under the process of formation (47,7% normalised importance in Model 1) or when groups were working on their final tasks (37.80% normalised importance in Model 3); meanwhile, the importance of perceived stress in explaining hair cortisol concentrations is low when the group is in a routine period of a group life-span (28.9% normalised importance in Model 2). Interpersonal cohesion (normalised importance 100% in Model 1 and 80.0% in Model 3) and task cohesion (normalised importance 78.6% in Model 2) were the most important predictors in the study area.

## Figures and Tables

**Table 1:** Demographic and anthropometric data for the groups at the beginning of their conscription training.

Characteristic	Value										
Group ID	1	2	3	4	5	6	7	8	9	10	11
Age (years),	20.90	21.20	20.75	19.63	20.13	20.10	20.10	20.70	20.67	20.50	19.30
(SD)	(1.287)	(1.619)	(1.815)	(1.061)	(1.885)	(1.524)	(1.524)	(1.418)	(2.291)	(1.716)	(0.823)
Education, *n* (%)											
Secondary	6 (60.0)	7 (70.0)	4 (33.3)	6 (75.0)	7 (87.5)	9 (90.0)	9 (90.0)	8 (80.0)	6 (66.7)	10 (100.0)	9 (90.0)
Vocational school	3 (30.0)	2 (20.0)	4 (33.3)	1 (12.5)	-	-	-	1 (10.0)	3 (33.3)	-	1 (10.0)
Higher education	1 (10.0)	1 (10.0)	4 (33.3)	1 (12.5)	1 (12.5)	1 (10.0)	1 (10.0)	1 (10.0)	-	-	-
Body mass index (kg/m^2^),	22.6504	25.3382	23.0042	24.4136	24.2505	22.2833	24.0421	22.8958	23.9878	23.1790	22.4325
(SD)	(1.615)	(4.457)	(2.852)	(2.203)	(4.283)	(2.822)	(3.474)	(2.537)	(3.184)	(1.575)	(1.794)

**Table 2:** Descriptive statistics for variables.

Variable	N	Minimum	Maximum	Mean	Std. Deviation	Variance	Statistic	
							Skewness	Kurtosis
**T1: at the beginning of training**								
Hair cortisol (COR_1)	112	0.010	1.340	0.575	0.25326	0.064	0.802	0.806
Perceived stress (PSS_1)	110	1	35	17.490	6.864	47.115	0.038	−0.141
Norm cohesion (CIN_1)	112	8	42	31.800	7.711	59.457	−0.790	−0.006
Interpersonal cohesion (CTE_1)	112	31	84	61.870	12.116	146.802	−0.360	−0.060
Task cohesion (CTS_1)	112	22	52	39.100	7.030	49.423	−0.407	−0.490
Valid N	110							
**T2: in the middle of training**								
Hair cortisol (COR_2)	106	0.050	1.160	0.523	0.21035	0.044	0.411	0.089
Perceived stress (PSS_2)	107	3	35	18.63	6.834	46.708	0.063	−0.429
Norm cohesion (CIN_2)	107	10	42	29.11	8.072	65.157	−0.227	−0.665
Interpersonal cohesion (CTE_2)	107	18	84	55.47	12.884	166.006	−0.208	−0.423
Task cohesion (CTS_2)	107	18	55	35.93	6.705	44.957	0.158	0.438
Valid N	106							
**T3: at the end of training**								
Hair cortisol (COR_3)	108	−0.03	0.98	0.460	0.201	0.041	0.180	−0.050
Perceived stress (PSS_3)	107	0	36	16.90	7.251	52.584	0.051	−0.287
Norm cohesion (CIN_3)	108	6	42	29.93	8.612	74.163	−0.581	−0.180
Interpersonal cohesion (CTE_3)	108	15	84	54.78	16.283	265.128	−0.134	−0.833
Task cohesion (CTS_3)	108	8	56	36.59	8.854	78.393	0.172	0.311
Valid N	107							

**Table 3:** Relationships between research variables.

	T1					T2					T3				
	COR1	CTE1	CTS1	CIN1	PSS1	COR2	CTE2	CTS2	CIN2	PSS2	COR3	CTE3	CTS3	CIN3	PSS3
COR_1,2,3_	1	−0.192 *	−0.221 *	−0.104	0.106	1	−0.190	−0.240 *	−0.143	0.208 *	1	−0.087	0.012	−0.096	0.176
CTE_1,2,3_	−0.192 *	1	0.739 **	0.568 **	−0.369 **	−0.190	1	0.628 **	0.592 **	−0.314 **	−0.087	1	0.794 **	0.781 **	−0.501 **
CTS_1,2,3_	−0.221 *	0.739 **	1	0.546 **	−0.499 **	−0.240 *	0.628 **	1	0.504 **	−0.396 **	0.012	0.794 **	1	0.672 **	−0.537 **
CIN_1,2,3_	−0.104	0.568 **	0.546 **	1	−0.499 **	−0.143	0.592 **	0.504 **	1	−0.377 **	−0.096	0.781 **	0.672 **	1	−0.506 **
PSS_1,2,3_	0.106	−0.369 **	−0.499 **	−0.499 **	1	0.208 *	−0.314 **	−0.396 **	−0.377 **	1	0.176	−0.501 **	−0.537 **	−0.506 **	1

Notes: Pearson’s correlation is significant: * at the 0.05 level (2-tailed); and ** at the 0.01 level (2-tailed). Abbreviations used: cortisol (COR), interpersonal cohesion (CTE), task cohesion (CTS), norm cohesion (CIN), perceived stress (PSS), numbers 1,2,3 indicate research periods.

**Table 4:** The normalised importance of independent variables in three models, by T1, T2, and T3 time points.

Model 1		Model 2		Model 3	
IV	NIMP	IV	NIMP	IV	NIMP
CTE1	100.0%	G_ID	100.0%	G_ID	100.00%
G_ID	87.4%	CTS2	78.6%	CTE3	80.00%
CTS1	59.2%	CTE2	32.4%	CIN3	47.60%
PSS1	47.7%	PSS2	28.9%	PSS3	37.80%
CIN1	23.4%	CIN2	16.0%	CTS3	22.10%

Notes: DT growing method= CRT; Model 1 = dependent variable COR_1 (at the beginning of conscription training); Model 2 = dependent variable COR_2 (in the middle of conscription training); Model 3 = dependent variable COR_3 (at the end of conscription training); NIMP= normalised importance. Abbreviations used: cortisol (COR), interpersonal cohesion (CTE), task cohesion (CTS), norm cohesion (CIN), perceived stress (PSS), numbers 1,2,3 indicate research periods.

## Data Availability

The data supporting reported results are archived in the National Open Access Research Data Archive (MIDAS) at www.midas.lt. (accessed on 16 November 2021).

## Appendix A

**Table A1:** Pairwise comparisons for paired differences in hair cortisol during three time points.

(I) Factor 1	(J) Factor 1	Mean Difference (I–J)	Std. Error	Sig.	95% Confidence Interval for Difference ^a^	
					Lower Bound	Upper Bound
COR_1	COR_2	0.041	0.023	0.222	−0.014	0.097
	COR_3	0.109 *	0.020	0.000	0.059	0.158
COR_2	COR_1	−0.041	0.023	0.222	−0.097	0.014
	COR_3	0.067 *	0.019	0.002	0.021	0.114
COR_3	COR_1	−0.109 *	0.020	0.000	−0.158	−0.059
	COR_2	−0.067 *	0.019	0.002	−0.114	−0.021

Notes: Based on estimated marginal means; * The mean difference is significant at the 0.05 level; ^a^ Adjustment for multiple comparisons: Bonferroni. Abbreviations used: cortisol (COR), numbers 1,2,3 indicate research periods.

**Table A2:** Design description for the constructed decision tree models.

Model Summary	Model 1	Model 2	Model 3
Specifications			
Growing Method	C&RT	C&RT	C&RT
Dependent Variable	COR_1	COR_2_	COR_3
Independent Variables	CTE1, CTS1, CIN1, PSS1, G ID	CTE2, CTS2, CIN2, PSS2, G ID	CTE3, CTS3, CIN3, PSS3, G ID
Validation	Yes	Yes	Yes
Maximum Tree Depth	5	5	5
Minimum Cases in Parent Node	15	15	15
Minimum Cases in Child Node	5	5	5
Results			
Independent Variables Included	G ID, CTS1, PSS1, CTE1	G ID, CTS2	G ID, CIN3, PSS3, CTE3
Number of Nodes	13	9	15
Number of Terminal Nodes	7	5	8
Depth	5	4	4

Notes: Model 1 relates to modelling with the first- time dataset (T1); Model 2 relates to modelling with the second time dataset (T2); and Model 3 relates to modelling with the third time dataset (T3). The split sample validation and the significance values were adjusted by Bonferroni method with min cases (15) in the parent node and min cases (5) in the child node for each of three models. Abbreviations used: cortisol (COR), interpersonal cohesion (CTE), task cohesion (CTS), norm cohesion (CIN), perceived stress (PSS), numbers 1,2,3 indicate research periods.

**Table A3:** Results description in table format for the designed C&RT Model 1.

Tree Table for Time Point T1									
Node	Mean	SD	N	Percent	Predicted Mean	Parent Node	Primary Independent Variable		
							Variable	Improvement	Split Values
0	0.5604	0.23561	107	100.0%	0.5604				
1	0.6960	0.27977	20	18.7%	0.6960	0	G_ID	0.004	2; 5
2	0.5292	0.21418	87	81.3%	0.5292	0	G_ID	0.004	1; 3; 4; 6; 7; 8; 9; 10; 11
3	0.7337	0.22919	6	5.6%	0.7337	2	CTS1	0.003	≤29.0
4	0.5141	0.20655	81	75.7%	0.5141	2	CTS1	0.003	>29.0
5	0.4991	0.19335	75	70.1%	0.4991	4	PSS1	0.002	≤25.5
6	0.7011	0.28988	6	5.6%	0.7011	4	PSS1	0.002	>25.5
7	0.4254	0.16513	26	24.3%	0.4254	5	CTE1	0.002	≤61.5
8	0.5383	0.19729	49	45.8%	0.5383	5	CTE1	0.002	>61.5
9	0.4722	0.15059	15	14.0%	0.4722	7	G_ID	0.001	1; 8; 9; 10
10	0.3616	0.16927	11	10.3%	0.3616	7	G_ID	0.001	4; 6; 11
11	0.6327	0.18788	20	18.7%	0.6327	8	CTE1	0.003	≤66.5
12	0.4731	0.17891	29	27.1%	0.4731	8	CTE1	0.003	>66.5

Notes: Growing method = CRT; value of five was used as a maximum tree depth (consisting of the number of total and terminal nodes), and fifteen was used as a minimum case in the parent node (relating to the number of levels below the root node); Dependent variable = COR_1. Abbreviations used: cortisol (COR), interpersonal cohesion (CTE), task cohesion (CTS), norm cohesion (CIN), perceived stress (PSS), numbers 1,2,3 indicate research periods.

**Table A4:** Results description in table format for the designed C&RT Model 2.

Tree Table for Time Point T2									
Node	Mean	SD	N	Percent	Predicted Mean	Parent Node	Primary Independent Variable		
							Variable	Improvement	Split Values
0	0.5173	0.21148	101	100.0%	0.5173				
1	0.5860	0.21609	48	47.5%	0.5860	0	G_ID	0.004	2; 5; 6; 7; 9
2	0.4550	0.18839	53	52.5%	0.4550	0	G_ID	0.004	1; 3; 4; 8; 10; 11
3	0.5217	0.21897	21	20.8%	0.5217	2	CTS2	0.002	≤34.5
4	0.4112	0.15366	32	31.7%	0.4112	2	CTS2	0.002	>34.5
5	0.4484	0.20346	15	14.9%	0.4484	3	G_ID	0.003	9; 1; 5; 7
6	0.7049	0.13957	6	5.9%	0.7049	3	G_ID	0.003	11; 8
7	0.3660	0.18571	8	7.9%	0.3660	5	CTS2	0.001	≤31.5
8	0.5425	0.19226	7	6.9%	0.5425	5	CTS2	0.001	>31.5

Notes: Growing method = CRT; value of five was used as a maximum tree depth (consisting of the number of total and terminal nodes), and fifteen was used as a minimum case in the parent node (relating to the number of levels below the root node). Dependent variable = COR_2. Abbreviations used: cortisol (COR), interpersonal cohesion (CTE), task cohesion (CTS), norm cohesion (CIN), perceived stress (PSS), numbers 1,2,3 indicate research periods.

**Table A5:** Results description in table format for the designed C&RT Model 3.

Tree Table for Time Point T3									
Node	Mean	SD	N	Percent	Predicted Mean	Parent Node	Primary Independent Variable		
							Variable	Improvement	Split Values
0	0.4522	0.19313	103	100.0%	0.4522				
1	0.3783	0.16614	53	51.5%	0.3783	0	G_ID	0.006	1; 2; 3; 8; 9; 10
2	0.5306	0.19019	50	48.5%	0.5306	0	G_ID	0.006	4; 5; 6; 7; 11
3	0.3148	0.16103	23	22.3%	0.3148	1	CIN3	0.002	≤31.5
4	0.4269	0.15548	30	29.1%	0.4269	1	CIN3	0.002	>31.5
5	0.4513	0.21312	21	20.4%	0.4513	2	PSS3	0.002	≤17.5
6	0.5880	0.15078	29	28.2%	0.5880	2	PSS3	0.002	>17.5
7	0.4252	0.10527	10	9.7%	0.4252	3	CTE3	0.002	≤41.0
8	0.2299	0.14579	13	12.6%	0.2299	3	CTE3	0.002	>41.0
9	0.5032	0.13572	15	14.6%	0.5032	4	G_ID	0.002	1; 8; 9
10	0.3507	0.13856	15	14.6%	0.3507	4	G_ID	0.002	2; 3; 10
11	0.3909	0.19484	16	15.5%	0.3909	5	CTE3	0.002	≤62.0
12	0.6447	0.15302	5	4.9%	0.6447	5	CTE3	0.002	>62.0
13	0.4796	0.24105	7	6.8%	0.4796	11	CIN3	0.001	≤31.5
14	0.3219	0.12398	9	8.7%	0.3219	11	CIN3	0.001	>31.5

Notes: Growing method = CRT; value of five was used as a maximum tree depth (consisting of the number of total and terminal nodes), and fifteen was used as a minimum case in the parent node (relating to the number of levels below the root node). Dependent variable = COR_3. Abbreviations used: cortisol (COR), interpersonal cohesion (CTE), task cohesion (CTS), norm cohesion (CIN), perceived stress (PSS), numbers 1,2,3 indicate research periods.
